# A Case of Optic Atrophy from ‘606’

**Published:** 1912-08

**Authors:** 


A Case of Optic Atrophy from '606'.—McNeil, Robb and Moll, (*Transvaal Med. Jour.,* May, 1912 report the case of a man 31 years old who had sores on the penis and glandular enlargements. Eighteen days after .6 gm. of Salvarsan were injected intramuscularly. Within a fortnight the sores had healed and he was placed on Hydr. cum Creta, 1-2 grain T. I. D. One month after he became blind in one eye and the diagnosis of optic neuritis was confirmed by an opththalmologist. No atrophy could be discovered in the other eye. They ascribe the atrophy to the effect of the Salvarsan.

(The case is instructive. The condition of the fundus oculi prior to the injection is not given so it may be assumed that it was not examined. More than 60,000 cases, we are informed, have received the drug without an authentic case of amaurosis. That it will hasten blindness when a spinal optic atrophy already exists seems certain; mercury will do the same. Mild degrees of optic neuritis are common in spyhlis and it is certainly unfair to charge a bad result to Salvarsan when it may well have developed as a result of the infective process.

In syphilitic iritis, neuroretinitis, choked disc, iritis papulosa, parenchymatous keratitis and chorioretinitis the effects of the drug have been found excellent and without untoward results. Ed.)

